# Bis(3,5-dimethyl-1*H*-pyrazole-κ*N* ^2^)bis­(3,3′′,5,5′′-tetra­methyl-[1,1′:3′,1′′-terphen­yl]-2′-carboxyl­ato-κ*O*)iron(II) dichloro­methane monosolvate

**DOI:** 10.1107/S1600536812015553

**Published:** 2012-04-18

**Authors:** Yeojin Jeon, Dharmalingam Sivanesan, Sungho Yoon

**Affiliations:** a Department of Bio & Nano Chemistry, College of Natural Sciences, Kookmin University, 861-1 Jeongneung-dong, Seongbuk-gu, Seoul 136-702, Republic of Korea

## Abstract

In the title compound, [Fe(C_23_H_21_O_2_)_2_(C_5_H_8_N_2_)_2_]·CH_2_Cl_2_, the Fe^2+^ cation is coordinated by the N atoms of two 3,5-dimethyl­pyrazole ligands and the carboxyl­ate O atoms from two tetra­methyl­terphenyl­carboxyl­ate ligands, forming an FeN_2_O_2_ polyhedron with a slightly distorted tetra­hedral coordination geometry. Intra­molecular N—H⋯O and C—H⋯O hydrogen-bonding inter­actions stabilize the mol­ecular conformation. The dihedral angles formed by the central benzene ring with the outer benzene rings of the terphenyl groups are 47.92 (8), 59.38 (8), 48.24 (8) and 52.37 (8)°. The dichloro­methane solvent mol­ecule inter­acts with the complex mol­ecule *via* a C—H⋯O hydrogen bond. In the crystal, centrosymmetrically related complex mol­ecules are linked into dimers through pairs of C—H⋯O hydrogen bonds.

## Related literature

For the synthesis of substituted terphenyl-based carboxyl­ate ligands, see: Saednya & Hart (1996 ▶); Du *et al.* (1986 ▶); Chen & Siegel (1994 ▶). For background to metal complexes with terphenyl-based carboxyl­ate and 3,5-dimethyl­pyrazole ligands, see: Hagadorn *et al.* (1998 ▶); Chakravorty *et al.* (2011 ▶); Kannan *et al.* (2011 ▶); Tolman & Que (2002 ▶); Zhang *et al.* (2007 ▶); Cheng *et al.* (1990 ▶).
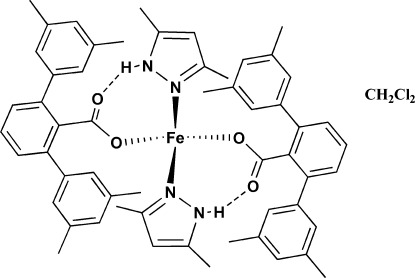


## Experimental

### 

#### Crystal data

[Fe(C_23_H_21_O_2_)_2_(C_5_H_8_N_2_)_2_]·CH_2_Cl_2_
*M* *_r_* = 991.84
Triclinic, 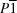

*a* = 12.363 (3) Å
*b* = 14.796 (3) Å
*c* = 16.735 (3) Å
α = 111.11 (3)°
β = 91.19 (3)°
γ = 109.06 (3)°
*V* = 2665.7 (13) Å^3^
*Z* = 2
Mo *K*α radiation
μ = 0.43 mm^−1^
*T* = 293 K
0.32 × 0.12 × 0.10 mm

#### Data collection

Bruker SMART CCD area-detector diffractometer
Absorption correction: multi-scan (*SADABS*; Bruker, 2000 ▶) *T*_min_ = 0.839, *T*_max_ = 1.0
20058 measured reflections
9561 independent reflections
8147 reflections with *I* > 2σ(*I*)
*R*_int_ = 0.018

#### Refinement

*R*[*F* ^2^ > 2σ(*F* ^2^)] = 0.053
*wR*(*F* ^2^) = 0.149
*S* = 1.07
9561 reflections
633 parameters
H atoms treated by a mixture of independent and constrained refinement
Δρ_max_ = 1.13 e Å^−3^
Δρ_min_ = −1.06 e Å^−3^

### 

Data collection: *SMART* (Bruker, 2000 ▶); cell refinement: *SAINT* (Bruker, 2000 ▶); data reduction: *SAINT*; program(s) used to solve structure: *SHELXS97* (Sheldrick, 2008 ▶); program(s) used to refine structure: *SHELXL97* (Sheldrick, 2008 ▶); molecular graphics: *SHELXTL* (Sheldrick, 2008 ▶); software used to prepare material for publication: *SHELXTL*.

## Supplementary Material

Crystal structure: contains datablock(s) I, global. DOI: 10.1107/S1600536812015553/rz2736sup1.cif

Structure factors: contains datablock(s) I. DOI: 10.1107/S1600536812015553/rz2736Isup2.hkl

Additional supplementary materials:  crystallographic information; 3D view; checkCIF report

## Figures and Tables

**Table 1:** Hydrogen-bond geometry (Å, °)

*D*—H⋯*A*	*D*—H	H⋯*A*	*D*⋯*A*	*D*—H⋯*A*
N4—H4⋯O4	0.84 (3)	1.95 (3)	2.744 (3)	158 (3)
N2—H2⋯O2	0.80 (3)	2.14 (3)	2.767 (3)	135 (3)
C6—H6*C*⋯O1	0.96	2.40	3.256 (4)	148
C46—H46⋯O3	0.93	2.46	3.055 (4)	122
C54—H54⋯O3	0.93	2.51	3.037 (3)	116
C55—H55*B*⋯O1	0.96	2.54	3.481 (3)	166
C58—H58*A*⋯O4	0.97	2.48	3.326 (5)	145
C3—H3⋯O4^i^	0.93	2.58	3.411 (5)	149

Symmetry code: (i) 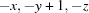.

**Fig. 1.**
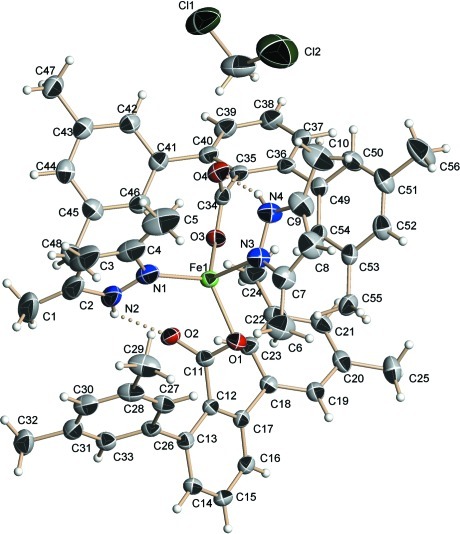
The molecular structure of the title compound, showing displacement ellipsoids drawn at the 50% probability level.

**Fig. 2.**
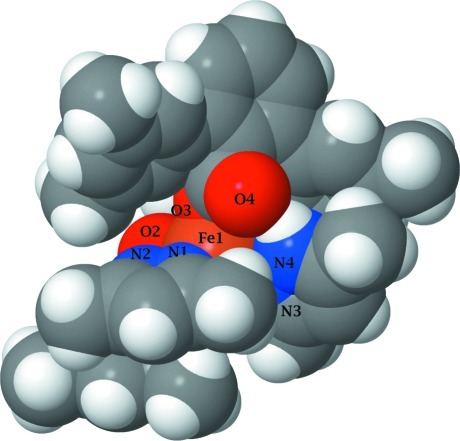
A space-filling representation of the title compound.

## supplementary crystallographic information

### Comment

The synthetic routes for sterically hindered terphenyl-based carboxylate ligands have been investigated (Saednya & Hart, 1996; Du *et al.*, 1986; Chen & Siegel, 1994). Recently, Fe^2+^ binuclear complexes of 3,3'',5,5''-tetramethyl-[1,1':3',1''-terphenyl]-2'-carboxylate have been synthesized for modeling dioxygen activation sites in diiron-containing proteins (Tolman & Que 2002). Four-coordinate Fe^2+^ and Co^2+^ metal complexes with a slightly distorted tetrahedral coordination geometry were reported with two 3,5-dimethylpyrazole and two benzoate ligands with less bulky substituents (Hagadorn *et al.*, 1998; Chakravorty *et al.*, 2011; Kannan *et al.*, 2011). Complexes with 3,5-dimethylpyrazole ligands have also been reported (Zhang *et al.*, 2007; Cheng *et al.*, 1990). Herein we report the structure of the tetrahedrally coordinated Fe^2+^ title complex with one dichloromethane molecule in the lattice.

In the title complex (Fig. 1), the iron(II) metal is coordinated by the N atoms of two 3,5-dimethylpyrazole ligands and the carboxylate O atoms of two tetramethyl-terphenyl carboxylate ligands in a slightly distorted tetrahedral geometry. In the carboxylate ligands, the dihedral angles formed by the central benzene ring (C12–C17 and C35–C40) with the outer benzene rings (C18–C23 and C26–C28/C30/C31/C33; C41–C46 and C40–C54) of the terphenyl groups are 47.92 (8), 59.38 (8), 48.24 (8) and 52.37 (8)°, respectively. The conformation of the complex is stabilized by intramolecular N—H···O and C—H···O hydrogen bonds (Table 1). A space filling model (Fig. 2) conveys the steric wall imposed by two terphenyl-based carboxylate and two 3,5-dimethylpyrazole ligands. The combined influence of the sterically hindered carboxylate ligands and the intramolecular hydrogen bonding interactions determines the binding mode of the carboxylate ligands as monodentate and stabilizes the coordination number of four of the metal ion. The dichloromethane molecule of crystallization interacts with the complex *via* a C—H···O hydrogen bond. In the crystal, centrosymmetrically related complex molecules are linked into dimers through pairs of C—H···O hydrogen bonds, generating a ring of *R*^2^_2_(16) motif.

### Experimental

Sodium 3,3'',5,5''-tetramethyl-[1,1':3',1''-terphenyl]-2'-carboxylate (0.206 g, 0.552 mmol) was mixed with Fe(BF_4_)_2_.6H_2_O (0.0930 g, 0.276 mmol) in tetrahydrofuran (10 mL) at room temperature. After overnight stirring, the white fine precipitate was filtered off and 3,5-dimethylpyrazole (0.0530 g, 0.276 mmol) was added. After three hours, tetrahydrofuran was removed under reduced pressure and colourless block-like crystals were obtained by crystallization from a dichloromethane/pentane (2:3 *v*/*v*) solution (yield 75%, 0.187 g). Anal. Calc. for FeC_56_H_58_O_4_N_4_: C, 74.16; H, 6.45; N, 6.18. Found: C, 73.80; H, 6.51; N, 6.02.

### Refinement

H atoms were placed at calculated positions and refined as riding with C–H(aromatic) = 0.95 Å, C–H(CH_3_) = 0.98 Å, and with *U*_iso_(H) = 1.2 *U*_eq_(C) or 1.5 *U*_eq_(C) for methyl groups. The N-bound H atoms were located in a difference Fourier map and refined isotropically.

### Crystal data

**Table:**

[Fe(C_23_H_21_O_2_)_2_(C_5_H_8_N_2_)_2_]·CH_2_Cl_2_	*Z* = 2
*M**_r_* = 991.84	*F*(000) = 1044
Triclinic, *P*1	*D*_x_ = 1.236 Mg m^−^^3^
Hall symbol: -P 1	Mo *K*α radiation, λ = 0.71073 Å
*a* = 12.363 (3) Å	Cell parameters from 1009 reflections
*b* = 14.796 (3) Å	θ = 3.1–27.7°
*c* = 16.735 (3) Å	µ = 0.43 mm^−^^1^
α = 111.11 (3)°	*T* = 293 K
β = 91.19 (3)°	Block, colorless
γ = 109.06 (3)°	0.32 × 0.12 × 0.10 mm
*V* = 2665.7 (13) Å^3^	

### Data collection

**Table:**

Bruker SMART CCD area-detector diffractometer	9561 independent reflections
Radiation source: fine-focus sealed tube	8147 reflections with *I* > 2σ(*I*)
Graphite monochromator	*R*_int_ = 0.018
φ and ω scans	θ_max_ = 25.3°, θ_min_ = 1.9°
Absorption correction: multi-scan (*SADABS*; Bruker, 2000)	*h* = −14→14
*T*_min_ = 0.839, *T*_max_ = 1.0	*k* = −17→17
20058 measured reflections	*l* = −20→19

### Refinement

**Table:**

Refinement on *F*^2^	Primary atom site location: structure-invariant direct methods
Least-squares matrix: full	Secondary atom site location: difference Fourier map
*R*[*F*^2^ > 2σ(*F*^2^)] = 0.053	Hydrogen site location: inferred from neighbouring sites
*wR*(*F*^2^) = 0.149	H atoms treated by a mixture of independent and constrained refinement
*S* = 1.07	*w* = 1/[σ^2^(*F*_o_^2^) + (0.0879*P*)^2^ + 1.2662*P*] where *P* = (*F*_o_^2^ + 2*F*_c_^2^)/3
9561 reflections	(Δ/σ)_max_ = 0.001
633 parameters	Δρ_max_ = 1.13 e Å^−^^3^
0 restraints	Δρ_min_ = −1.06 e Å^−^^3^

### Special details

**Table:**

Geometry. All esds (except the esd in the dihedral angle between two l.s. planes) are estimated using the full covariance matrix. The cell esds are taken into account individually in the estimation of esds in distances, angles and torsion angles; correlations between esds in cell parameters are only used when they are defined by crystal symmetry. An approximate (isotropic) treatment of cell esds is used for estimating esds involving l.s. planes.
Refinement. Refinement of F^2^ against ALL reflections. The weighted R-factor wR and goodness of fit S are based on F^2^, conventional R-factors R are based on F, with F set to zero for negative F^2^. The threshold expression of F^2^ > 2sigma(F^2^) is used only for calculating R-factors(gt) etc. and is not relevant to the choice of reflections for refinement. R-factors based on F^2^ are statistically about twice as large as those based on F, and R- factors based on ALL data will be even larger.

### Fractional atomic coordinates and isotropic or equivalent isotropic displacement parameters (Å^2^)

**Table:**

	*x*	*y*	*z*	*U*_iso_*/*U*_eq_	
Fe1	0.20939 (3)	0.51707 (2)	0.21446 (2)	0.02958 (12)	
O3	0.30443 (15)	0.66135 (12)	0.23370 (11)	0.0348 (4)	
O1	0.29491 (14)	0.45286 (13)	0.26950 (11)	0.0351 (4)	
O2	0.36189 (15)	0.45601 (13)	0.14937 (11)	0.0361 (4)	
O4	0.16310 (14)	0.72328 (13)	0.23166 (11)	0.0401 (4)	
C34	0.2667 (2)	0.73433 (17)	0.24544 (14)	0.0303 (5)	
C11	0.36139 (19)	0.43290 (16)	0.21395 (15)	0.0294 (5)	
N1	0.1156 (2)	0.43116 (16)	0.09177 (15)	0.0444 (5)	
C17	0.5361 (2)	0.42978 (17)	0.29198 (14)	0.0299 (5)	
C18	0.57352 (19)	0.54266 (18)	0.34720 (15)	0.0304 (5)	
C12	0.43746 (19)	0.37735 (17)	0.22818 (14)	0.0277 (5)	
N3	0.08654 (18)	0.52658 (17)	0.30033 (15)	0.0404 (5)	
C27	0.1936 (2)	0.21195 (18)	0.12314 (17)	0.0369 (5)	
H27	0.1820	0.2437	0.1796	0.044*	
C40	0.4078 (2)	0.89183 (18)	0.22863 (16)	0.0343 (5)	
C23	0.5838 (2)	0.61643 (18)	0.31164 (16)	0.0342 (5)	
H23	0.5639	0.5950	0.2521	0.041*	
C49	0.3161 (2)	0.84844 (18)	0.43131 (15)	0.0326 (5)	
C35	0.35406 (19)	0.84372 (17)	0.28261 (15)	0.0297 (5)	
C54	0.3374 (2)	0.76141 (18)	0.43030 (14)	0.0311 (5)	
H54	0.3837	0.7355	0.3922	0.037*	
C20	0.6418 (2)	0.6807 (2)	0.49019 (16)	0.0392 (6)	
C36	0.3690 (2)	0.90032 (18)	0.37248 (15)	0.0318 (5)	
C13	0.4061 (2)	0.27119 (17)	0.17776 (14)	0.0297 (5)	
C16	0.6018 (2)	0.37335 (19)	0.30439 (15)	0.0334 (5)	
H16	0.6692	0.4076	0.3450	0.040*	
N4	0.04781 (19)	0.60714 (19)	0.32059 (16)	0.0433 (5)	
C33	0.3208 (2)	0.16675 (18)	0.02217 (16)	0.0363 (5)	
H33	0.3945	0.1679	0.0110	0.044*	
C19	0.6032 (2)	0.57645 (19)	0.43686 (15)	0.0336 (5)	
H19	0.5968	0.5278	0.4612	0.040*	
C26	0.3044 (2)	0.21535 (17)	0.10658 (16)	0.0320 (5)	
C15	0.5684 (2)	0.26825 (19)	0.25767 (16)	0.0356 (5)	
H15	0.6112	0.2315	0.2687	0.043*	
C41	0.3931 (2)	0.83493 (18)	0.13292 (16)	0.0345 (5)	
C37	0.4345 (2)	1.00550 (19)	0.40654 (16)	0.0388 (6)	
H37	0.4431	1.0438	0.4657	0.047*	
C22	0.6234 (2)	0.72150 (19)	0.36390 (18)	0.0392 (6)	
C14	0.4715 (2)	0.21733 (18)	0.19445 (15)	0.0338 (5)	
H14	0.4497	0.1464	0.1627	0.041*	
C45	0.3935 (2)	0.6881 (2)	0.00764 (18)	0.0412 (6)	
C53	0.2906 (2)	0.71222 (19)	0.48535 (15)	0.0344 (5)	
N2	0.1702 (3)	0.39093 (19)	0.02638 (15)	0.0512 (6)	
C21	0.6517 (2)	0.7522 (2)	0.45271 (17)	0.0411 (6)	
H21	0.6779	0.8225	0.4880	0.049*	
C30	0.1192 (2)	0.1149 (2)	−0.02760 (19)	0.0471 (7)	
H30	0.0570	0.0816	−0.0727	0.057*	
C55	0.3162 (2)	0.6194 (2)	0.48315 (17)	0.0426 (6)	
H55A	0.3864	0.6415	0.5220	0.064*	
H55B	0.3250	0.5810	0.4253	0.064*	
H55C	0.2534	0.5760	0.5008	0.064*	
C28	0.1002 (2)	0.16175 (19)	0.0564 (2)	0.0438 (6)	
C43	0.3497 (2)	0.8277 (2)	−0.01151 (17)	0.0403 (6)	
C46	0.4087 (2)	0.74075 (19)	0.09713 (17)	0.0375 (6)	
H46	0.4297	0.7122	0.1335	0.045*	
C39	0.4754 (2)	0.99731 (19)	0.26556 (18)	0.0414 (6)	
H39	0.5131	1.0298	0.2303	0.050*	
C42	0.3634 (2)	0.8778 (2)	0.07789 (17)	0.0380 (6)	
H42	0.3527	0.9410	0.1015	0.046*	
C31	0.2285 (2)	0.11647 (19)	−0.04582 (17)	0.0419 (6)	
C50	0.2496 (2)	0.8881 (2)	0.49064 (17)	0.0431 (6)	
H50	0.2355	0.9466	0.4921	0.052*	
C52	0.2241 (2)	0.7537 (2)	0.54303 (17)	0.0432 (6)	
H52	0.1921	0.7214	0.5799	0.052*	
C7	0.0312 (2)	0.4704 (2)	0.34426 (19)	0.0468 (6)	
C38	0.4876 (2)	1.05429 (19)	0.35319 (18)	0.0442 (6)	
H38	0.5311	1.1250	0.3764	0.053*	
C44	0.3637 (2)	0.7328 (2)	−0.04499 (17)	0.0418 (6)	
H44	0.3528	0.6978	−0.1049	0.050*	
C8	−0.0422 (3)	0.5161 (3)	0.3913 (2)	0.0557 (8)	
H8	−0.0904	0.4922	0.4269	0.067*	
C2	0.1004 (4)	0.3431 (2)	−0.0506 (2)	0.0722 (11)	
C47	0.3212 (3)	0.8779 (3)	−0.06921 (19)	0.0544 (8)	
H47A	0.3913	0.9153	−0.0845	0.082*	
H47B	0.2838	0.9250	−0.0389	0.082*	
H47C	0.2704	0.8253	−0.1209	0.082*	
C32	0.2471 (3)	0.0662 (2)	−0.13746 (18)	0.0581 (8)	
H32A	0.3238	0.1029	−0.1444	0.087*	
H32B	0.1912	0.0681	−0.1769	0.087*	
H32C	0.2381	−0.0047	−0.1496	0.087*	
C29	−0.0194 (2)	0.1596 (2)	0.0747 (3)	0.0619 (9)	
H29A	−0.0228	0.2274	0.0882	0.093*	
H29B	−0.0359	0.1397	0.1230	0.093*	
H29C	−0.0756	0.1105	0.0245	0.093*	
C51	0.2041 (2)	0.8426 (2)	0.54731 (18)	0.0483 (7)	
C9	−0.0298 (2)	0.6024 (2)	0.3749 (2)	0.0525 (7)	
C6	0.0509 (3)	0.3745 (3)	0.3385 (2)	0.0633 (9)	
H6A	−0.0016	0.3162	0.2907	0.095*	
H6B	0.0377	0.3639	0.3913	0.095*	
H6C	0.1292	0.3815	0.3298	0.095*	
C4	0.0078 (3)	0.4087 (2)	0.0543 (2)	0.0614 (9)	
C3	−0.0040 (4)	0.3534 (3)	−0.0343 (3)	0.0834 (14)	
H3	−0.0702	0.3281	−0.0748	0.100*	
C25	0.6735 (3)	0.7153 (2)	0.58695 (17)	0.0545 (7)	
H25A	0.7539	0.7585	0.6046	0.082*	
H25B	0.6595	0.6556	0.6012	0.082*	
H25C	0.6270	0.7538	0.6165	0.082*	
C1	0.1433 (5)	0.2950 (3)	−0.1326 (2)	0.1081 (19)	
H1A	0.2237	0.3060	−0.1197	0.162*	
H1B	0.1342	0.3262	−0.1723	0.162*	
H1C	0.0995	0.2218	−0.1583	0.162*	
C48	0.4112 (3)	0.5862 (2)	−0.0304 (2)	0.0589 (8)	
H48A	0.3459	0.5364	−0.0737	0.088*	
H48B	0.4186	0.5618	0.0146	0.088*	
H48C	0.4804	0.5953	−0.0564	0.088*	
C24	0.6358 (3)	0.8004 (2)	0.3240 (2)	0.0558 (8)	
H24A	0.6236	0.8600	0.3648	0.084*	
H24B	0.5793	0.7703	0.2726	0.084*	
H24C	0.7121	0.8212	0.3093	0.084*	
C10	−0.0849 (3)	0.6826 (3)	0.4048 (3)	0.0726 (10)	
H10A	−0.0290	0.7493	0.4132	0.109*	
H10B	−0.1119	0.6847	0.4586	0.109*	
H10C	−0.1491	0.6653	0.3620	0.109*	
C56	0.1346 (4)	0.8883 (3)	0.6133 (3)	0.0804 (12)	
H56A	0.1864	0.9430	0.6636	0.121*	
H56B	0.0845	0.8352	0.6298	0.121*	
H56C	0.0888	0.9158	0.5884	0.121*	
C5	−0.0779 (3)	0.4417 (3)	0.1061 (3)	0.0847 (13)	
H5A	−0.1034	0.4004	0.1398	0.127*	
H5B	−0.1431	0.4326	0.0679	0.127*	
H5C	−0.0429	0.5135	0.1441	0.127*	
Cl1	0.15706 (12)	1.02798 (9)	0.20616 (8)	0.1043 (4)	
Cl2	0.05816 (15)	0.95928 (15)	0.33896 (13)	0.1414 (6)	
C58	0.1625 (4)	0.9602 (3)	0.2718 (3)	0.0840 (12)	
H58A	0.1522	0.8892	0.2352	0.101*	
H58B	0.2385	0.9915	0.3071	0.101*	
H2	0.235 (3)	0.393 (2)	0.0360 (19)	0.039 (8)*	
H4	0.074 (3)	0.653 (2)	0.3006 (19)	0.044 (8)*	

### Atomic displacement parameters (Å^2^)

**Table:**

	*U*^11^	*U*^22^	*U*^33^	*U*^12^	*U*^13^	*U*^23^
Fe1	0.02992 (19)	0.02477 (18)	0.0333 (2)	0.00762 (14)	0.00257 (14)	0.01248 (14)
O3	0.0421 (9)	0.0232 (8)	0.0374 (9)	0.0088 (7)	0.0063 (7)	0.0124 (7)
O1	0.0349 (9)	0.0347 (9)	0.0382 (9)	0.0156 (7)	0.0082 (7)	0.0139 (7)
O2	0.0401 (9)	0.0352 (9)	0.0374 (9)	0.0152 (8)	0.0016 (7)	0.0175 (7)
O4	0.0326 (9)	0.0368 (9)	0.0453 (10)	0.0054 (7)	−0.0051 (8)	0.0167 (8)
C34	0.0366 (13)	0.0280 (12)	0.0251 (11)	0.0080 (10)	0.0024 (9)	0.0122 (9)
C11	0.0290 (11)	0.0210 (11)	0.0317 (12)	0.0045 (9)	−0.0015 (9)	0.0075 (9)
N1	0.0455 (13)	0.0317 (11)	0.0472 (13)	0.0071 (10)	−0.0123 (10)	0.0126 (10)
C17	0.0327 (12)	0.0308 (12)	0.0269 (11)	0.0110 (10)	0.0055 (9)	0.0124 (9)
C18	0.0266 (11)	0.0302 (12)	0.0328 (12)	0.0099 (9)	0.0037 (9)	0.0108 (10)
C12	0.0302 (11)	0.0277 (11)	0.0265 (11)	0.0104 (9)	0.0052 (9)	0.0119 (9)
N3	0.0335 (11)	0.0412 (12)	0.0519 (13)	0.0138 (9)	0.0131 (10)	0.0232 (10)
C27	0.0353 (13)	0.0238 (11)	0.0470 (14)	0.0073 (10)	0.0041 (11)	0.0117 (10)
C40	0.0340 (12)	0.0304 (12)	0.0381 (13)	0.0096 (10)	−0.0019 (10)	0.0150 (10)
C23	0.0338 (12)	0.0337 (12)	0.0343 (12)	0.0115 (10)	0.0004 (10)	0.0130 (10)
C49	0.0317 (12)	0.0294 (12)	0.0276 (11)	0.0071 (10)	−0.0030 (9)	0.0049 (9)
C35	0.0297 (11)	0.0248 (11)	0.0354 (12)	0.0100 (9)	−0.0011 (9)	0.0125 (9)
C54	0.0315 (12)	0.0314 (12)	0.0269 (11)	0.0103 (10)	0.0005 (9)	0.0084 (9)
C20	0.0315 (12)	0.0427 (14)	0.0353 (13)	0.0138 (11)	0.0051 (10)	0.0058 (11)
C36	0.0326 (12)	0.0283 (12)	0.0330 (12)	0.0120 (10)	−0.0042 (9)	0.0096 (10)
C13	0.0314 (12)	0.0297 (11)	0.0283 (11)	0.0096 (10)	0.0062 (9)	0.0128 (9)
C16	0.0347 (12)	0.0381 (13)	0.0287 (12)	0.0139 (11)	0.0017 (10)	0.0137 (10)
N4	0.0304 (11)	0.0451 (13)	0.0607 (15)	0.0159 (10)	0.0142 (10)	0.0252 (12)
C33	0.0355 (13)	0.0288 (12)	0.0404 (13)	0.0086 (10)	0.0012 (10)	0.0116 (10)
C19	0.0298 (12)	0.0367 (13)	0.0331 (12)	0.0118 (10)	0.0044 (10)	0.0122 (10)
C26	0.0339 (12)	0.0227 (11)	0.0377 (13)	0.0081 (9)	0.0003 (10)	0.0121 (10)
C15	0.0425 (14)	0.0382 (13)	0.0363 (13)	0.0219 (11)	0.0061 (11)	0.0191 (11)
C41	0.0307 (12)	0.0324 (12)	0.0391 (13)	0.0049 (10)	0.0033 (10)	0.0183 (10)
C37	0.0428 (14)	0.0308 (13)	0.0358 (13)	0.0116 (11)	−0.0087 (11)	0.0073 (10)
C22	0.0320 (13)	0.0326 (13)	0.0504 (15)	0.0110 (10)	0.0024 (11)	0.0140 (11)
C14	0.0416 (13)	0.0266 (11)	0.0354 (13)	0.0142 (10)	0.0062 (10)	0.0127 (10)
C45	0.0383 (14)	0.0375 (14)	0.0471 (15)	0.0096 (11)	0.0160 (12)	0.0188 (12)
C53	0.0345 (12)	0.0368 (13)	0.0257 (11)	0.0076 (10)	0.0006 (10)	0.0103 (10)
N2	0.0705 (19)	0.0394 (13)	0.0365 (13)	0.0188 (13)	−0.0135 (12)	0.0085 (10)
C21	0.0336 (13)	0.0320 (13)	0.0463 (15)	0.0113 (11)	0.0020 (11)	0.0030 (11)
C30	0.0413 (15)	0.0315 (13)	0.0550 (17)	0.0038 (11)	−0.0160 (12)	0.0113 (12)
C55	0.0498 (16)	0.0469 (15)	0.0371 (14)	0.0167 (13)	0.0092 (12)	0.0235 (12)
C28	0.0331 (13)	0.0258 (12)	0.0668 (18)	0.0052 (10)	−0.0011 (12)	0.0169 (12)
C43	0.0325 (13)	0.0520 (16)	0.0415 (14)	0.0139 (12)	0.0089 (11)	0.0247 (12)
C46	0.0362 (13)	0.0373 (13)	0.0435 (14)	0.0111 (11)	0.0087 (11)	0.0226 (11)
C39	0.0423 (14)	0.0321 (13)	0.0482 (15)	0.0051 (11)	−0.0003 (12)	0.0215 (12)
C42	0.0372 (13)	0.0377 (13)	0.0426 (14)	0.0123 (11)	0.0083 (11)	0.0205 (11)
C31	0.0491 (15)	0.0291 (12)	0.0394 (14)	0.0077 (11)	−0.0046 (12)	0.0107 (11)
C50	0.0440 (15)	0.0332 (13)	0.0450 (15)	0.0157 (12)	0.0034 (12)	0.0057 (11)
C52	0.0425 (15)	0.0431 (15)	0.0337 (13)	0.0063 (12)	0.0099 (11)	0.0114 (11)
C7	0.0369 (14)	0.0471 (16)	0.0552 (17)	0.0088 (12)	0.0139 (12)	0.0235 (13)
C38	0.0466 (15)	0.0252 (12)	0.0492 (16)	0.0022 (11)	−0.0111 (12)	0.0121 (11)
C44	0.0374 (14)	0.0493 (15)	0.0356 (13)	0.0094 (12)	0.0110 (11)	0.0181 (12)
C8	0.0406 (16)	0.0610 (19)	0.0636 (19)	0.0103 (14)	0.0233 (14)	0.0282 (16)
C2	0.118 (3)	0.0394 (17)	0.0440 (18)	0.0205 (19)	−0.0268 (19)	0.0077 (14)
C47	0.0555 (18)	0.079 (2)	0.0466 (16)	0.0351 (17)	0.0146 (14)	0.0341 (16)
C32	0.073 (2)	0.0507 (17)	0.0374 (15)	0.0153 (16)	−0.0071 (14)	0.0089 (13)
C29	0.0342 (15)	0.0421 (16)	0.097 (3)	0.0081 (13)	−0.0001 (15)	0.0192 (17)
C51	0.0462 (16)	0.0459 (16)	0.0420 (15)	0.0135 (13)	0.0136 (12)	0.0073 (12)
C9	0.0299 (14)	0.0577 (18)	0.0629 (19)	0.0144 (13)	0.0139 (13)	0.0165 (15)
C6	0.064 (2)	0.0564 (19)	0.083 (2)	0.0184 (16)	0.0330 (18)	0.0438 (18)
C4	0.0531 (18)	0.0385 (15)	0.076 (2)	0.0004 (14)	−0.0293 (16)	0.0205 (15)
C3	0.097 (3)	0.0468 (19)	0.077 (3)	0.0054 (19)	−0.055 (2)	0.0137 (18)
C25	0.0584 (18)	0.0549 (18)	0.0328 (14)	0.0142 (15)	0.0027 (13)	0.0032 (13)
C1	0.208 (6)	0.068 (3)	0.0365 (19)	0.056 (3)	−0.015 (3)	0.0044 (17)
C48	0.075 (2)	0.0495 (17)	0.0571 (19)	0.0268 (16)	0.0313 (17)	0.0215 (15)
C24	0.0583 (18)	0.0365 (15)	0.070 (2)	0.0121 (13)	−0.0026 (15)	0.0228 (14)
C10	0.0482 (18)	0.074 (2)	0.097 (3)	0.0307 (17)	0.0301 (18)	0.026 (2)
C56	0.089 (3)	0.067 (2)	0.083 (3)	0.035 (2)	0.047 (2)	0.017 (2)
C5	0.0388 (18)	0.087 (3)	0.126 (4)	0.0121 (18)	−0.019 (2)	0.049 (3)
Cl1	0.1176 (9)	0.0743 (7)	0.1066 (9)	0.0258 (6)	−0.0435 (7)	0.0302 (6)
Cl2	0.1312 (12)	0.1640 (15)	0.1702 (16)	0.0944 (12)	0.0593 (11)	0.0716 (13)
C58	0.077 (3)	0.067 (2)	0.114 (3)	0.036 (2)	−0.001 (2)	0.034 (2)

### Geometric parameters (Å, º)

**Table:**

Fe1—O3	1.9727 (17)	C30—H30	0.9300
Fe1—O1	2.0286 (17)	C55—H55A	0.9600
Fe1—N1	2.058 (2)	C55—H55B	0.9600
Fe1—N3	2.116 (2)	C55—H55C	0.9600
O3—C34	1.266 (3)	C28—C29	1.509 (4)
O1—C11	1.273 (3)	C43—C44	1.383 (4)
O2—C11	1.245 (3)	C43—C42	1.387 (4)
O4—C34	1.242 (3)	C43—C47	1.511 (4)
C34—C35	1.509 (3)	C46—H46	0.9300
C11—C12	1.501 (3)	C39—C38	1.379 (4)
N1—C4	1.343 (4)	C39—H39	0.9300
N1—N2	1.354 (4)	C42—H42	0.9300
C17—C16	1.400 (3)	C31—C32	1.504 (4)
C17—C12	1.400 (3)	C50—C51	1.379 (4)
C17—C18	1.491 (3)	C50—H50	0.9300
C18—C23	1.394 (3)	C52—C51	1.394 (4)
C18—C19	1.397 (3)	C52—H52	0.9300
C12—C13	1.401 (3)	C7—C8	1.396 (4)
N3—C7	1.337 (3)	C7—C6	1.486 (4)
N3—N4	1.361 (3)	C38—H38	0.9300
C27—C28	1.386 (4)	C44—H44	0.9300
C27—C26	1.392 (3)	C8—C9	1.362 (5)
C27—H27	0.9300	C8—H8	0.9300
C40—C35	1.394 (3)	C2—C3	1.370 (6)
C40—C39	1.396 (4)	C2—C1	1.499 (6)
C40—C41	1.491 (3)	C47—H47A	0.9600
C23—C22	1.388 (3)	C47—H47B	0.9600
C23—H23	0.9300	C47—H47C	0.9600
C49—C54	1.390 (3)	C32—H32A	0.9600
C49—C50	1.391 (4)	C32—H32B	0.9600
C49—C36	1.493 (3)	C32—H32C	0.9600
C35—C36	1.406 (3)	C29—H29A	0.9600
C54—C53	1.394 (3)	C29—H29B	0.9600
C54—H54	0.9300	C29—H29C	0.9600
C20—C19	1.383 (4)	C51—C56	1.515 (4)
C20—C21	1.388 (4)	C9—C10	1.495 (4)
C20—C25	1.511 (4)	C6—H6A	0.9600
C36—C37	1.386 (3)	C6—H6B	0.9600
C13—C14	1.393 (3)	C6—H6C	0.9600
C13—C26	1.494 (3)	C4—C3	1.389 (5)
C16—C15	1.375 (3)	C4—C5	1.478 (6)
C16—H16	0.9300	C3—H3	0.9300
N4—C9	1.337 (4)	C25—H25A	0.9600
N4—H4	0.84 (3)	C25—H25B	0.9600
C33—C26	1.389 (3)	C25—H25C	0.9600
C33—C31	1.390 (4)	C1—H1A	0.9600
C33—H33	0.9300	C1—H1B	0.9600
C19—H19	0.9300	C1—H1C	0.9600
C15—C14	1.381 (4)	C48—H48A	0.9600
C15—H15	0.9300	C48—H48B	0.9600
C41—C46	1.385 (4)	C48—H48C	0.9600
C41—C42	1.394 (3)	C24—H24A	0.9600
C37—C38	1.389 (4)	C24—H24B	0.9600
C37—H37	0.9300	C24—H24C	0.9600
C22—C21	1.388 (4)	C10—H10A	0.9600
C22—C24	1.511 (4)	C10—H10B	0.9600
C14—H14	0.9300	C10—H10C	0.9600
C45—C44	1.383 (4)	C56—H56A	0.9600
C45—C46	1.392 (4)	C56—H56B	0.9600
C45—C48	1.502 (4)	C56—H56C	0.9600
C53—C52	1.385 (4)	C5—H5A	0.9600
C53—C55	1.496 (4)	C5—H5B	0.9600
N2—C2	1.343 (4)	C5—H5C	0.9600
N2—H2	0.80 (3)	Cl1—C58	1.747 (4)
C21—H21	0.9300	Cl2—C58	1.728 (5)
C30—C31	1.386 (4)	C58—H58A	0.9700
C30—C28	1.388 (4)	C58—H58B	0.9700
			
O3—Fe1—O1	111.85 (7)	C45—C46—H46	119.5
O3—Fe1—N1	114.09 (9)	C38—C39—C40	121.4 (2)
O1—Fe1—N1	119.33 (8)	C38—C39—H39	119.3
O3—Fe1—N3	106.17 (8)	C40—C39—H39	119.3
O1—Fe1—N3	96.59 (8)	C43—C42—C41	121.0 (2)
N1—Fe1—N3	106.18 (10)	C43—C42—H42	119.5
C34—O3—Fe1	125.58 (15)	C41—C42—H42	119.5
C11—O1—Fe1	99.69 (14)	C30—C31—C33	118.6 (3)
O4—C34—O3	125.2 (2)	C30—C31—C32	120.7 (3)
O4—C34—C35	117.4 (2)	C33—C31—C32	120.7 (3)
O3—C34—C35	117.3 (2)	C51—C50—C49	121.6 (3)
O2—C11—O1	121.8 (2)	C51—C50—H50	119.2
O2—C11—C12	121.1 (2)	C49—C50—H50	119.2
O1—C11—C12	117.09 (19)	C53—C52—C51	121.7 (2)
C4—N1—N2	105.6 (3)	C53—C52—H52	119.1
C4—N1—Fe1	135.5 (2)	C51—C52—H52	119.1
N2—N1—Fe1	118.65 (18)	N3—C7—C8	109.5 (3)
C16—C17—C12	118.3 (2)	N3—C7—C6	121.3 (3)
C16—C17—C18	118.9 (2)	C8—C7—C6	129.2 (3)
C12—C17—C18	122.8 (2)	C39—C38—C37	119.6 (2)
C23—C18—C19	118.7 (2)	C39—C38—H38	120.2
C23—C18—C17	121.8 (2)	C37—C38—H38	120.2
C19—C18—C17	119.5 (2)	C43—C44—C45	122.2 (2)
C17—C12—C13	120.6 (2)	C43—C44—H44	118.9
C17—C12—C11	120.8 (2)	C45—C44—H44	118.9
C13—C12—C11	118.6 (2)	C9—C8—C7	106.8 (3)
C7—N3—N4	105.3 (2)	C9—C8—H8	126.6
C7—N3—Fe1	135.96 (19)	C7—C8—H8	126.6
N4—N3—Fe1	118.77 (16)	N2—C2—C3	106.1 (3)
C28—C27—C26	120.7 (3)	N2—C2—C1	121.3 (4)
C28—C27—H27	119.6	C3—C2—C1	132.6 (4)
C26—C27—H27	119.6	C43—C47—H47A	109.5
C35—C40—C39	118.6 (2)	C43—C47—H47B	109.5
C35—C40—C41	122.2 (2)	H47A—C47—H47B	109.5
C39—C40—C41	119.2 (2)	C43—C47—H47C	109.5
C22—C23—C18	120.9 (2)	H47A—C47—H47C	109.5
C22—C23—H23	119.6	H47B—C47—H47C	109.5
C18—C23—H23	119.6	C31—C32—H32A	109.5
C54—C49—C50	118.6 (2)	C31—C32—H32B	109.5
C54—C49—C36	120.0 (2)	H32A—C32—H32B	109.5
C50—C49—C36	121.4 (2)	C31—C32—H32C	109.5
C40—C35—C36	120.5 (2)	H32A—C32—H32C	109.5
C40—C35—C34	121.0 (2)	H32B—C32—H32C	109.5
C36—C35—C34	118.0 (2)	C28—C29—H29A	109.5
C49—C54—C53	121.3 (2)	C28—C29—H29B	109.5
C49—C54—H54	119.4	H29A—C29—H29B	109.5
C53—C54—H54	119.4	C28—C29—H29C	109.5
C19—C20—C21	118.5 (2)	H29A—C29—H29C	109.5
C19—C20—C25	120.4 (3)	H29B—C29—H29C	109.5
C21—C20—C25	121.1 (2)	C50—C51—C52	118.4 (2)
C37—C36—C35	119.2 (2)	C50—C51—C56	121.0 (3)
C37—C36—C49	119.9 (2)	C52—C51—C56	120.5 (3)
C35—C36—C49	120.9 (2)	N4—C9—C8	106.5 (3)
C14—C13—C12	119.1 (2)	N4—C9—C10	121.0 (3)
C14—C13—C26	119.7 (2)	C8—C9—C10	132.6 (3)
C12—C13—C26	121.2 (2)	C7—C6—H6A	109.5
C15—C16—C17	121.2 (2)	C7—C6—H6B	109.5
C15—C16—H16	119.4	H6A—C6—H6B	109.5
C17—C16—H16	119.4	C7—C6—H6C	109.5
C9—N4—N3	112.0 (2)	H6A—C6—H6C	109.5
C9—N4—H4	128 (2)	H6B—C6—H6C	109.5
N3—N4—H4	120 (2)	N1—C4—C3	109.4 (4)
C26—C33—C31	120.8 (2)	N1—C4—C5	121.2 (3)
C26—C33—H33	119.6	C3—C4—C5	129.4 (3)
C31—C33—H33	119.6	C2—C3—C4	107.0 (3)
C20—C19—C18	121.4 (2)	C2—C3—H3	126.5
C20—C19—H19	119.3	C4—C3—H3	126.5
C18—C19—H19	119.3	C20—C25—H25A	109.5
C33—C26—C27	119.3 (2)	C20—C25—H25B	109.5
C33—C26—C13	119.4 (2)	H25A—C25—H25B	109.5
C27—C26—C13	121.2 (2)	C20—C25—H25C	109.5
C16—C15—C14	120.0 (2)	H25A—C25—H25C	109.5
C16—C15—H15	120.0	H25B—C25—H25C	109.5
C14—C15—H15	120.0	C2—C1—H1A	109.5
C46—C41—C42	119.0 (2)	C2—C1—H1B	109.5
C46—C41—C40	121.7 (2)	H1A—C1—H1B	109.5
C42—C41—C40	119.2 (2)	C2—C1—H1C	109.5
C36—C37—C38	120.6 (2)	H1A—C1—H1C	109.5
C36—C37—H37	119.7	H1B—C1—H1C	109.5
C38—C37—H37	119.7	C45—C48—H48A	109.5
C21—C22—C23	118.9 (2)	C45—C48—H48B	109.5
C21—C22—C24	121.0 (2)	H48A—C48—H48B	109.5
C23—C22—C24	120.1 (2)	C45—C48—H48C	109.5
C15—C14—C13	120.7 (2)	H48A—C48—H48C	109.5
C15—C14—H14	119.7	H48B—C48—H48C	109.5
C13—C14—H14	119.7	C22—C24—H24A	109.5
C44—C45—C46	118.4 (2)	C22—C24—H24B	109.5
C44—C45—C48	121.1 (3)	H24A—C24—H24B	109.5
C46—C45—C48	120.5 (3)	C22—C24—H24C	109.5
C52—C53—C54	118.3 (2)	H24A—C24—H24C	109.5
C52—C53—C55	121.8 (2)	H24B—C24—H24C	109.5
C54—C53—C55	119.9 (2)	C9—C10—H10A	109.5
C2—N2—N1	111.9 (3)	C9—C10—H10B	109.5
C2—N2—H2	127 (2)	H10A—C10—H10B	109.5
N1—N2—H2	121 (2)	C9—C10—H10C	109.5
C22—C21—C20	121.7 (2)	H10A—C10—H10C	109.5
C22—C21—H21	119.2	H10B—C10—H10C	109.5
C20—C21—H21	119.2	C51—C56—H56A	109.5
C31—C30—C28	121.6 (2)	C51—C56—H56B	109.5
C31—C30—H30	119.2	H56A—C56—H56B	109.5
C28—C30—H30	119.2	C51—C56—H56C	109.5
C53—C55—H55A	109.5	H56A—C56—H56C	109.5
C53—C55—H55B	109.5	H56B—C56—H56C	109.5
H55A—C55—H55B	109.5	C4—C5—H5A	109.5
C53—C55—H55C	109.5	C4—C5—H5B	109.5
H55A—C55—H55C	109.5	H5A—C5—H5B	109.5
H55B—C55—H55C	109.5	C4—C5—H5C	109.5
C27—C28—C30	118.8 (3)	H5A—C5—H5C	109.5
C27—C28—C29	120.4 (3)	H5B—C5—H5C	109.5
C30—C28—C29	120.8 (3)	Cl2—C58—Cl1	113.0 (2)
C44—C43—C42	118.4 (2)	Cl2—C58—H58A	109.0
C44—C43—C47	122.0 (2)	Cl1—C58—H58A	109.0
C42—C43—C47	119.6 (3)	Cl2—C58—H58B	109.0
C41—C46—C45	121.0 (2)	Cl1—C58—H58B	109.0
C41—C46—H46	119.5	H58A—C58—H58B	107.8

### Hydrogen-bond geometry (Å, º)

**Table:**

*D*—H···*A*	*D*—H	H···*A*	*D*···*A*	*D*—H···*A*
N4—H4···O4	0.84 (3)	1.95 (3)	2.744 (3)	158 (3)
N2—H2···O2	0.80 (3)	2.14 (3)	2.767 (3)	135 (3)
C6—H6*C*···O1	0.96	2.40	3.256 (4)	148
C46—H46···O3	0.93	2.46	3.055 (4)	122
C54—H54···O3	0.93	2.51	3.037 (3)	116
C55—H55*B*···O1	0.96	2.54	3.481 (3)	166
C58—H58*A*···O4	0.97	2.48	3.326 (5)	145
C3—H3···O4^i^	0.93	2.58	3.411 (5)	149

Symmetry code: (i) −*x*, −*y*+1, −*z*.
